# Current Studies on the Hypoxic Tumor Microenvironment in Thyroid Cancer: From Molecular Mechanisms to Clinical Therapeutic Perspectives

**DOI:** 10.3390/biomedicines14051126

**Published:** 2026-05-16

**Authors:** Xuejiao Peng, Li Ma, Weiqin Chang

**Affiliations:** Department of Thyroid Surgery, The Second Hospital of Jilin University, Changchun 130041, China; pengxj23@mails.jlu.edu.cn (X.P.);

**Keywords:** hypoxia-inducible factor-1α, tumor microenvironment, dedifferentiation, radioiodine resistance, epithelial–mesenchymal transition

## Abstract

Hypoxia is a hallmark feature of solid tumors and is increasingly recognized as an important factor in tumor progression, aggressiveness, and therapeutic resistance. In the tumor microenvironment, hypoxia is associated with genetic instability, abnormal angiogenesis, metabolic reprogramming, and crosstalk with oncogenic signaling pathways, thereby potentially enhancing tumor invasiveness and metastatic potential. Furthermore, hypoxia may impair the sensitivity of tumor cells to conventional therapies and contribute to treatment resistance. This article reviews current evidence on the role of hypoxia in thyroid cancer, focusing on its biological effects, clinical implications, and therapeutic relevance. Available studies suggest that hypoxia may affect thyroid cancer progression and treatment tolerance by modulating hypoxia-inducible factor (HIF) signaling, epithelial–mesenchymal transition (EMT), angiogenesis, metabolic adaptation, cancer stem-like properties, extracellular matrix remodeling, and stress-adaptive responses. However, the strength of evidence varies across these pathways, and many hypoxia-targeted strategies remain under preclinical investigation. Approaches such as HIF inhibition, redifferentiation therapy, and vascular modulation may offer potential therapeutic directions for advanced and refractory thyroid cancer. Given the marked heterogeneity of thyroid cancer, further thyroid cancer-specific studies are needed to clarify the prognostic and therapeutic significance of hypoxia.

## 1. Introduction

Thyroid cancer is the most common endocrine malignancy worldwide, and its incidence has been rising steadily in recent decades [1,2,3,4]. Papillary thyroid cancer (PTC), follicular thyroid cancer (FTC), and anaplastic thyroid cancer (ATC) arise from follicular epithelial cells (thyrocytes), whereas medullary thyroid cancer (MTC) originates from parafollicular C cells. PTC is the most common subtype, and together with FTC accounts for approximately 90% of all thyroid cancers as differentiated thyroid cancer (DTC). MTC and ATC account for 2–8% and 1–2% of thyroid cancers, respectively [5]. DTC usually has a good prognosis, with a 5-year survival rate exceeding 90%. ATC is the most aggressive subtype with the worst prognosis, while MTC has a prognosis between DTC and ATC.

A distinguishing feature of most solid tumors, including thyroid cancers, is the presence of a hypoxic tumor microenvironment (TME), particularly in rapidly growing lesions [6]. Tumor hypoxia develops when oxygen demand exceeds vascular supply and may also be exacerbated by anticancer therapies that impair tumor perfusion. Under hypoxic conditions, tumor cells undergo a series of adaptive changes, including activation of hypoxia-inducible factors (HIFs), promotion of angiogenesis, metabolic reprogramming, enhancement of metastatic potential, and reduction in sensitivity to therapy [7]. In thyroid cancer, HIF-1α and HIF-2α are increasingly recognized as important mediators linking hypoxia to tumor progression, dedifferentiation, and treatment resistance. However, despite growing evidence linking hypoxia to thyroid cancer progression, the underlying molecular mechanisms remain incompletely understood, and therapeutic strategies targeting the hypoxic TME are still under investigation.

This review summarizes current evidence on the role of hypoxia in thyroid cancer, with a focus on its effects on tumor biology, progression, and treatment resistance. We also explore emerging strategies for targeting the hypoxic TME, including HIF inhibition, metabolic reprogramming, and vascular regulation. By integrating current findings, this review aims to elucidate the biological and clinical relevance of hypoxia in thyroid cancer and to highlight potential directions for future therapeutic development in advanced and refractory disease.

To prepare this narrative review, we searched PubMed, Web of Science, and Google Scholar for studies published over the past two decades. Search terms included combinations of “thyroid cancer”, “poorly differentiated thyroid cancer”, “hypoxia”, “HIF-1α”, “HIF-2α”, “angiogenesis”, “epithelial–mesenchymal transition”, “stemness”, “dedifferentiation”, “radioiodine resistance”, and “tumor microenvironment”. Priority was given to English-language original studies and clinically relevant reviews focused on thyroid cancer. When direct thyroid cancer evidence was limited, representative findings from other solid tumors were included to provide a mechanistic or therapeutic context; these findings are identified in the text as extrapolative rather than thyroid-specific.

## 2. Hypoxia in the Tumor Microenvironment: General Concepts

The cellular response to hypoxia is primarily regulated by HIFs, particularly HIF-1α and HIF-2α. Under hypoxic conditions, these factors are stabilized and function as transcriptional regulators of a broad range of target genes involved in cellular adaptation, survival, and tumor progression. HIFs are composed of α and β subunits that form a heterodimeric complex in the nucleus, bind to hypoxia-responsive elements (HREs) in target genes, and activate transcriptional programs involved in cellular adaptation to hypoxia [8,9].

HIF signaling is regulated by both oxygen availability and tumor-associated genetic alterations in the tumor microenvironment [10]. Under normoxic conditions, prolyl hydroxylase domain (PHD) enzymes use oxygen to hydroxylate key proline residues on HIF-α subunits, thereby enabling recognition by the von Hippel–Lindau (VHL) tumor suppressor complex and subsequent proteasomal degradation. Under hypoxic conditions, PHD enzyme activity is inhibited, leading to stabilization and accumulation of HIF-α in the cytoplasm, followed by nuclear translocation and heterodimerization with HIF-1β. In addition, non-hypoxic stimuli, including growth factors, cytokines, and oncogenic signals, can enhance HIF-1α expression through the PI3K/AKT and MAPK pathways, thereby promoting tumor adaptation and progression [11].

Activation of HIF signaling induces metabolic reprogramming, shifting cellular energy production from oxidative phosphorylation to aerobic glycolysis (the Warburg effect) [12]. This process is accompanied by increased expression of glucose transporters such as glucose transporter 1 (GLUT1) and glucose transporter 3 (GLUT3), as well as key glycolytic enzymes including pyruvate kinase M2 (PKM2), thereby supporting tumor growth under hypoxic conditions [13]. In addition, hypoxia stimulates the expression of vascular endothelial growth factor (VEGF) and other pro-angiogenic mediators, promoting angiogenesis [14]. However, the newly formed tumor vasculature is often structurally and functionally abnormal, further exacerbating regional hypoxia [15,16].

Hypoxia can promote EMT and the acquisition of cancer stem cell (CSC)-like properties, thereby enhancing invasion, metastasis, and recurrence [17]. In addition, hypoxia can reshape the tumor immune microenvironment by upregulating immunosuppressive pathways such as PD-L1 expression, recruiting regulatory T cells and myeloid-derived suppressor cells, and impairing cytotoxic T-cell activity [18]. Collectively, these effects may reduce the efficacy of chemotherapy, radiotherapy, and immunotherapy [16,19].

Elevated HIF-1α expression has been reported in a variety of malignancies, including breast cancer [20], oral cancer [21], cervical cancer [22], gastric cancer [23], prostate cancer [24], pancreatic cancer [25], lung cancer [26], and glioma [27]. Many of the hypoxia-associated mechanisms described above are broadly shared across solid tumors. However, the extent to which these processes have been specifically validated in thyroid cancer varies, and the following sections therefore distinguish, where possible, between general solid-tumor concepts and thyroid cancer-specific evidence.

## 3. Hypoxia in Thyroid Cancer

### 3.1. Expression of HIF-1α and HIF-2α in Thyroid Cancer

HIF-1α and HIF-2α expression levels have been reported to be significantly higher in PTC tissues than in normal thyroid tissues [28]. In tissue-based studies, increased HIF-1α expression has been associated with adverse clinicopathological features in PTC, including larger tumor size, lymph node metastasis, advanced tumor stage, capsular invasion, and extrathyroidal extension [28]. Consistent with these findings, a recent study from our group further showed that HIF-1α expression was significantly higher in PTC tissues than in nodular goiter and normal thyroid tissues. Such elevated HIF-1α expression was associated with central cervical lymph node metastasis in PTC [29]. Similarly, elevated HIF-2α expression has also been linked to lymph node metastasis, tumor stage, capsular invasion, and extrathyroidal extension [28]. These findings suggest that increased HIF-1α and HIF-2α expression may be associated with adverse clinicopathological features in PTC. However, the available evidence is not entirely consistent across studies. For example, Zhang et al. [30] reported that HIF-1α was overexpressed in PTC tissues and associated with capsular invasion and poor prognosis, but did not observe a significant association with tumor size or lymph node metastasis. Such discrepancies may reflect differences in cohort size, patient composition, or study design. Therefore, although HIF-1α and HIF-2α show promise as candidate biomarkers in PTC, further validation in larger and more standardized clinical cohorts is still needed.

In addition to tissue studies, preclinical evidence also supports a functional role for HIF-1α in thyroid cancer. Jin et al. [31] demonstrated high HIF-1α expression in BCPAP thyroid cancer cells and showed that HIF-1α inhibition reduced HIF-1α expression and suppressed tumor cell proliferation, migration, and invasiveness. These findings support the biological relevance of HIF signaling in thyroid cancer, although they remain limited to experimental models.

Elevated HIF-1α expression has also been reported in FTC [32], MTC [33], and ATC [34], although the expression patterns appear to vary across histological subtypes. Burrows et al. [34] found that HIF-1α expression increased with tumor aggressiveness, with the highest and most diffuse expression observed in poorly differentiated thyroid cancer (PDTC) and ATC. In contrast, PTC and FTC showed more focal staining patterns. Klaus et al. [32] also reported HIF-1α expression in a subset of FTC cases. However, evidence in non-PTC subtypes remains comparatively limited, and the biological significance of HIF-1α expression across these histological subtypes remains to be further investigated.

### 3.2. HIF-1α Facilitates EMT and Metastasis

EMT refers to the process by which epithelial cancer cells acquire mesenchymal characteristics, resulting in reduced cell–cell adhesion, loss of polarity, and increased motility. In thyroid cancer, available experimental evidence suggests that hypoxia promotes EMT at least in part through HIF-1α activation and induction of EMT-related genes [35]. Under hypoxic conditions, HIF-1α has been reported to upregulate key EMT-associated transcription factors, including Twist, Snail, and Slug, thereby altering cell morphology and enhancing invasive potential [36]. Additional thyroid cancer-specific studies further support this mechanism. For example, hypoxia was shown to induce IL-11 secretion through HIF-1α, which subsequently activated the PI3K/Akt/GSK3β signaling pathway to promote EMT and metastatic behavior in ATC cells [36]. Yang et al. [37] also demonstrated that the SIRT6/HIF-1α axis promotes PTC progression through EMT induction.

Consistent with these observations, hypoxic thyroid cancer cells exhibit characteristic EMT-associated phenotypic changes, suggesting that HIF-1α-mediated EMT may contribute to metastatic progression. In addition to EMT-related effects, HIF-1α may interact with broader microenvironmental programs that support tumor dissemination [38]. However, current evidence is derived mainly from experimental models, and the clinical significance of hypoxia-driven EMT in thyroid cancer remains to be further defined.

### 3.3. Hypoxia-Driven Angiogenesis in Thyroid Cancer

Angiogenesis is a key process supporting tumor growth and metastatic spread, particularly in highly vascular thyroid tumors, and is mediated largely by VEGF, a major downstream target of HIF-1α [39]. In a hypoxic microenvironment, HIF-1α upregulation increases VEGF production, thereby stimulating neovascularization and supporting tumor progression. In thyroid cancer, available studies suggest that VEGF expression is closely associated with HIF-1α activity. Elevated HIF-1α levels have been positively correlated with increased VEGF expression in PTC and ATC, supporting a role for hypoxia-associated angiogenic signaling in thyroid cancer [34,40]. Consistent with this, HIF-1α silencing has been reported to downregulate VEGF expression and suppress the invasive and metastatic capacity of thyroid cancer cells [41]. Together, these findings support an important role for the HIF-1α/VEGF axis in hypoxia-associated angiogenesis in thyroid cancer.

HIF-1α may also promote angiogenesis indirectly through the regulation of non-coding RNAs. Under hypoxic conditions, HIF-1α has been reported to regulate microRNA-181a (miR-181a), which promotes angiogenesis by downregulating GATA6 expression [42]. GATA6 is a transcription factor involved in cellular differentiation and vascular regulatory programs, and its suppression may favor a pro-angiogenic phenotype in thyroid cancer. These findings further suggest that hypoxia-driven angiogenesis in thyroid cancer is controlled not only by canonical HIF-1α/VEGF signaling, but also by broader transcriptional and post-transcriptional regulatory networks.

### 3.4. Hypoxia-Induced Metabolic Reprogramming in Thyroid Cancer

Metabolic reprogramming is a hallmark of malignant tumors, enabling cancer cells to sustain rapid proliferation and survival under environmental stress [43]. Under hypoxic conditions, thyroid cancer cells undergo a shift from oxidative phosphorylation toward glycolysis, a process driven in part by HIF-1α and other hypoxia-responsive regulators [44]. Although glycolysis is less efficient than oxidative phosphorylation in terms of ATP yield, it can rapidly provide metabolic intermediates and energy to support tumor growth. In several cancers, hypoxia and glycolysis form a reinforcing loop, in which enhanced glycolytic flux activates oncogenic pathways, while hypoxia further increases cellular dependence on glucose metabolism [12,13]. Consistent with this, HIF signaling upregulates multiple glycolysis-related genes, including GLUT1, lactate dehydrogenase A (LDHA), and pyruvate dehydrogenase kinase (PDK), thereby promoting a Warburg-like metabolic phenotype [45,46].

Aberrant GLUT expression has been reported in multiple malignancies, including breast cancer [47], pancreatic cancer [48], gastric cancer [49], and colorectal cancer [50], where it is often associated with tumor progression and prognosis. In thyroid cancer, available studies suggest that hypoxia and oncogenic alterations cooperate to increase GLUT expression and glucose uptake. GLUT1 and GLUT3, both high-affinity glucose transporters, appear to be particularly important in this process and are influenced by the hypoxic tumor microenvironment [51]. Thyroid cancer-specific studies have reported GLUT1 expression in PTC and ATC, with more pronounced expression in PDTC [52]. Similarly, Nahm et al. reported elevated GLUT1 and GLUT3 expression across a cohort of patients with PTC, FTC, MTC, and ATC, with the highest GLUT1 expression observed in ATC [53]. Moreover, GLUT1 mRNA and protein levels were significantly higher in PTC cell lines than in normal thyroid cells [54]. Collectively, these findings suggest that increased GLUT expression may be associated with metabolic adaptation and lower differentiation status in thyroid cancer. However, whether targeting glucose transport directly can provide therapeutic benefit in thyroid cancer remains to be clarified.

Increased glucose uptake is accompanied by altered expression of key glycolytic enzymes, including pyruvate kinase and lactate dehydrogenase [13]. Among these, PKM2 plays an important regulatory role in the final step of glycolysis and has been implicated in cancer metabolic reprogramming and progression [55]. PKM2 is upregulated in multiple malignancies and has been associated with poor prognosis in several tumor types [56,57,58,59]. In thyroid cancer, PKM2 expression has been reported to be elevated across multiple histological subtypes compared with benign thyroid lesions, with the highest levels observed in ATC [60]. Feng et al. [61] further showed that PKM2 overexpression was associated with BRAFV600E mutation, advanced clinical stage, and lymph node metastasis. In addition, available evidence suggests that PKM2 upregulation may contribute to treatment resistance in thyroid cancer. NF-κB signaling has been linked to radioiodine resistance in PTC, and Wang et al. [62] reported that HIF-1α promoted resistance-related phenotypes in PTC by regulating PKM2 and NF-κB expression. These findings suggest that hypoxia-driven metabolic reprogramming may contribute to metabolic adaptation and treatment resistance in thyroid cancer. However, the clinical and therapeutic significance of these metabolic alterations in thyroid cancer remains to be further defined.

### 3.5. Hypoxia and HIF Signaling Maintain Cancer Stem Cell Features in Thyroid Cancer

Thyroid cancer exhibits substantial intratumoral heterogeneity, with differences in cellular phenotype, biological behavior, and oncogenic alterations contributing to variable proliferative capacity, differentiation status, and therapeutic response [63,64]. Cancer stem cells (CSCs), which possess self-renewal and multilineage differentiation potential, are thought to play an important role in tumor growth, heterogeneity, recurrence, and treatment resistance. In thyroid cancer, CSC-associated features have been implicated in disease progression and therapeutic failure, particularly in aggressive subtypes such as ATC [65,66,67,68]. Available evidence further suggests that CSCs preferentially reside in specialized hypoxic niches, where HIF signaling helps maintain stemness [46,69].

In thyroid cancer, experimental studies indicate that hypoxia promotes CSC survival and stem-like characteristics within hypoxic and stromal niches [70]. Under these conditions, HIF signaling appears to enrich the CSC fraction and sustain stem-like properties. Mahkamova et al. [71] reported that hypoxia enriched thyroid CSC populations in PTC, FTC, and ATC cell lines. Similarly, Lan et al. [72] showed that HIF-1α overexpression induced EMT in FTC cells and promoted a more aggressive, ATC-like phenotype. Additional studies suggest that hypoxic niches and HIF signaling may also contribute to reduced therapeutic sensitivity [70]. However, current evidence remains derived mainly from experimental models, and the clinical significance of hypoxia-driven CSC maintenance in thyroid cancer remains to be clarified.

### 3.6. HIF-1α Promotes the Dedifferentiation of Thyroid Cancer

The development of thyroid cancer is a multistep process involving the accumulation of molecular alterations that may drive progression from well-differentiated thyroid cancer to PDTC or ATC [73,74]. Clinicopathological observations support this evolutionary relationship. A substantial proportion of patients with ATC have a history of thyroid nodules or PTC, and PTC foci are frequently identified in ATC tissue specimens. In addition, shared molecular alterations between differentiated and anaplastic components suggest that some differentiated thyroid cancers may undergo dedifferentiation during disease progression [75,76]. Available evidence suggests that hypoxia may contribute to this process by promoting molecular and transcriptional changes associated with loss of differentiation.

This issue is of particular clinical importance in ATC, which accounts for the majority of thyroid cancer-related deaths. At diagnosis, most patients with ATC already have locally advanced or metastatic disease, and prognosis remains extremely poor despite multimodal treatment, with a median overall survival of only 3–6 months [77]. Moreover, 20–60% of patients with ATC have a history of DTC or concurrent thyroid cancer, most commonly PTC [78,79]. These observations support the concept that dedifferentiation is a clinically relevant event in thyroid cancer progression and highlight the potential relevance of hypoxia in this process.

Thyroid cancer-specific studies further suggest that HIF-1α may be involved in hypoxia-associated dedifferentiation. Powell et al. [80] identified miR-210 as a hypoxia-responsive microRNA that was significantly upregulated in thyroid cancer cells, with higher expression in ATC than in PTC. Because miR-210 expression was inversely associated with differentiation status, it may represent both a marker and a mediator of hypoxia-related dedifferentiation. In addition, Ma et al. [81] reported that ATC exhibited a higher hypoxia score than PDTC, and that higher hypoxia scores were associated with lower differentiation status, greater genetic complexity, and increased tumor aggressiveness. PDTC also showed higher hypoxia scores than well-differentiated tumors and normal thyroid tissue. Collectively, these findings suggest that hypoxia and HIF-1α signaling may contribute to dedifferentiation in thyroid cancer. However, further mechanistic and clinical studies are required to determine whether targeting hypoxia can meaningfully alter the course of dedifferentiated thyroid cancer.

### 3.7. Hypoxia-Induced Radioiodine (RAI) Resistance and Broader Therapeutic Resistance in Thyroid Cancer

In a hypoxic TME, cancer cells activate HIF signaling, which promotes metabolic reprogramming and enhances multiple survival pathways, including glycolysis, angiogenesis, and multidrug resistance-related programs, thereby contributing to therapeutic resistance [81,82,83,84]. In thyroid cancer, available evidence suggests that hypoxia is an important contributor to treatment resistance, particularly in PDTC and ATC, where sustained HIF activation appears to enhance tumor cell survival and adaptive stress responses.

RAI therapy remains a cornerstone of treatment for DTC and depends on preserved expression of the sodium/iodide symporter (NIS) to mediate iodine uptake. However, some patients develop RAI-refractory disease, especially in PDTC or ATC, which is associated with poor prognosis and limited therapeutic options. Thyroid cancer-specific studies suggest that hypoxia contributes to RAI resistance through HIF-1α-dependent signaling pathways [84,85]. For example, pharmacological inhibition of HIF-1α has been reported to restore NIS expression and partially resensitize thyroid cancer cells to RAI [86]. Multiple downstream pathways have also been implicated in this process. Wang et al. [62] showed that HIF-1α contributed to RAI-resistant phenotypes in PTC through the PKM2/NF-κB axis, supporting tumor cell survival and inflammatory signaling. In addition, HIF signaling has been reported to reduce NIS protein abundance through the HIF-1α/β-catenin pathway [87]. Song et al. [88] further found that hypoxia-induced YAP activation increased GLUT expression while decreasing NIS expression, thereby promoting RAI resistance. Collectively, these findings indicate that hypoxia-driven RAI resistance in thyroid cancer is mediated by complex metabolic, inflammatory, and differentiation-related mechanisms. These pathways represent an important component of hypoxia-associated therapeutic resistance in thyroid cancer, with shared functional consequences despite distinct molecular drivers.

In addition to its effects on RAI response, HIF-1α has been reported to suppress the expression of thyroid-specific genes, including NIS, thyroid-stimulating hormone receptor (TSHR), thyroglobulin (TG), and thyroid peroxidase (TPO), thereby impairing iodine uptake and contributing to loss of thyroid differentiation [86,88]. This mechanism may be particularly relevant in RAI-refractory DTC, which shares some molecular features with dedifferentiated thyroid tumors and often exhibits elevated HIF-1α activity. Beyond radioiodine resistance, hypoxia may also influence the response to radiotherapy and other treatments through additional mechanisms, including extracellular matrix remodeling and stress-adaptive pathways such as the heat shock response and unfolded protein response. However, direct evidence for these broader resistance mechanisms in thyroid cancer remains limited. Overall, current findings support a role for HIF signaling in radioiodine resistance and related adaptive stress responses in thyroid cancer, although further studies are required to clarify its therapeutic relevance.

### 3.8. HIF-1α, Lymph Node Metastasis, and Prognosis in Thyroid Cancer

PTC is frequently associated with lymph node metastasis (LNM), which can occur even in relatively early-stage disease and may contribute to recurrence and shortened disease-free survival [89]. Because small primary tumors and occult nodal involvement can limit the sensitivity of imaging-based assessment, there is continued interest in identifying biomarkers that may improve risk stratification and prognostic evaluation in PTC. In this context, available studies suggest that elevated HIF-1α expression may have prognostic relevance in PTC. Zhao et al. [90] reported that HIF-1α overexpression was associated with a higher risk of LNM in PTC and poorer prognosis in thyroid cancer. Their findings further suggested that HIF-1α expression may have prognostic value for disease-free interval in PTC.

Additional evidence supports a functional link between HIF-1α and metastatic behavior in thyroid cancer. For example, Natalie et al. [91] found that the PI3K inhibitor GDC-0941 suppressed metastatic progression in FTC models, at least in part through inhibition of HIF-1α. Taken together, these findings support a possible association between HIF-1α and adverse clinicopathological outcomes in thyroid cancer. However, further validation across subtypes is needed before HIF-related markers can be applied in clinical prognostic assessment.

### 3.9. Influence of the BRAFV600E on the Regulation of HIF-1α Expression

Although the BRAFV600E is observed in several cancer types, its interaction with hypoxia-related signaling appears to be particularly relevant in thyroid cancer, especially PTC. Available evidence suggests that crosstalk between oncogenic BRAF signaling and HIF-1α-mediated hypoxic adaptation may contribute to several aggressive features of thyroid cancer, including dedifferentiation, lymph node metastasis, and reduced sensitivity to radioiodine therapy.

Thyroid cancer-specific studies further suggest that BRAFV600E can influence HIF-1α expression. Zerilli et al. [92] reported that the BRAFV600E increased HIF-1α expression in PTC cells, whereas silencing of BRAFV600E reduced HIF-1α levels. Additional thyroid cancer evidence has also suggested a link between BRAFV600E status and hypoxia-related protein expression [93]. Although thyroid cancer-specific mechanistic evidence remains limited, studies in other BRAF-driven tumors, particularly melanoma, support the possibility that oncogenic BRAF signaling may enhance HIF-1α expression and alter hypoxia-adaptive functions through MAPK-dependent and post-translational mechanisms [94,95].

### 3.10. Hypoxia-Associated Extracellular Matrix Remodeling and Stiffness in Thyroid Cancer

In addition to regulating intracellular transcriptional programs, hypoxia can reshape the biomechanical properties of the TME. In a variety of solid tumors, hypoxia-driven extracellular matrix (ECM) remodeling, collagen cross-linking, and matrix stiffening have been implicated in tumor invasion, drug penetration, and response to radiotherapy [19,96,97]. However, direct evidence linking hypoxia to stromal stiffening in thyroid cancer remains limited. Growing evidence suggests that thyroid cancer progression is accompanied by stromal remodeling, fibroblast activation, collagen deposition, and altered tumor–stromal interactions [98,99]. In particular, fibroblast-mediated collagen remodeling and upregulation of COL1A1 and LOX have been associated with aggressive thyroid cancer phenotypes. Meanwhile, increased peritumoral stiffness has been linked to cancer-associated fibroblast (CAF) activation, ECM remodeling, LNM, and poor prognosis in PTC [98,100]. Collectively, these findings suggest that hypoxia-associated ECM remodeling and biomechanical stiffening may influence thyroid cancer progression and the tumor microenvironment, although this role has not yet been fully defined.

### 3.11. Heat Shock Response and Unfolded Protein Response Under Hypoxia

In addition to HIF-dependent metabolic and invasive programs, hypoxia can impose proteotoxic stress on cancer cells, leading to the accumulation of misfolded proteins and endoplasmic reticulum (ER) stress. Under hypoxic conditions, tumor cells activate the unfolded protein response (UPR), a conserved signaling network involving the PERK, ATF6, and IRE1 pathways, which helps restore protein homeostasis under stress [101,102]. In response to persistent hypoxic stress, tumor cells may also initiate the heat shock response by upregulating heat shock proteins (HSPs), including HSP70 and HSP90, which stabilize unfolded proteins and promote proper protein folding [103,104,105].

These adaptive responses support tumor cell survival under harsh microenvironmental conditions and have also been associated with radiotherapy tolerance and treatment failure in a range of solid tumors [106,107]. Mechanistically, UPR and HSP-related pathways can inhibit apoptosis and enhance tolerance to radiation- or cytotoxic-drug-induced stress. Although direct evidence in thyroid cancer remains limited, increased expression of HSPs and UPR-associated genes has been observed in more aggressive thyroid cancer subtypes [103,108,109]. These findings suggest that hypoxia-induced protein homeostasis pathways may represent an underrecognized stress-adaptive mechanism in thyroid malignancies, with possible relevance to treatment resistance.

### 3.12. Additional Molecular Mechanisms by Which Hypoxia Regulates Thyroid Cancer Behavior

Under hypoxic conditions, thyroid cancer cells have been reported to release extracellular vesicles containing microRNAs and proteins that may contribute to EMT and angiogenesis [42,110,111]. Additional thyroid cancer-specific studies have identified other hypoxia-responsive pathways that may influence tumor progression. For example, Chen et al. [112] reported that FGF11 was significantly upregulated in hypoxic TPC-1 cells, suggesting that HIF-1α may promote thyroid cancer progression through an HIF-1α/FGF11 feedback loop. Song [113] further described a HIF-1α/TERT-associated mechanism that induced autophagy through the mTOR pathway and promoted PTC progression under hypoxic stress. Although these mechanisms require further validation, they suggest that hypoxia may regulate thyroid cancer behavior through additional transcriptional, vesicle-mediated, and stress-adaptive pathways beyond the major mechanisms discussed above.

**Figure 1:**
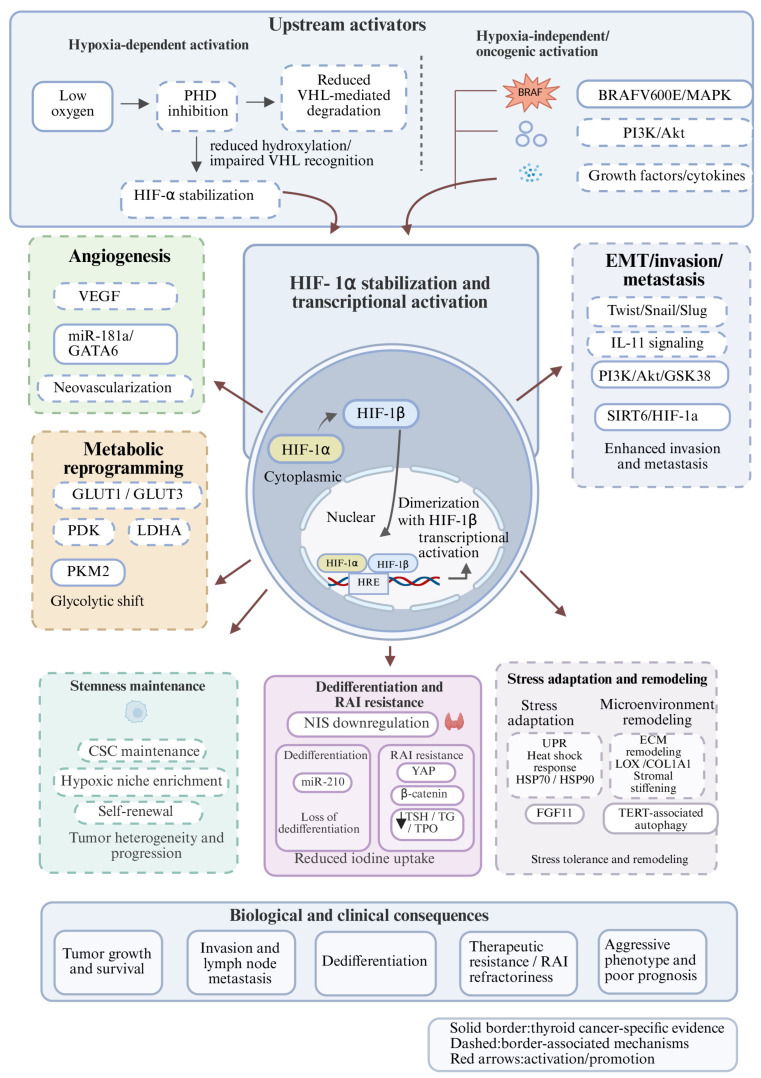
Integrated schematic of hypoxia/HIF-1α signaling in thyroid cancer. Hypoxia-dependent and hypoxia-independent signals converge on HIF-1α stabilization and activation in thyroid cancer. Reduced oxygen availability inhibits PHD activity, impairs VHL-mediated degradation, and promotes HIF-1α stabilization, while oncogenic and non-hypoxic inputs, including BRAFV600E/MAPK, PI3K/Akt, and growth factors/cytokines, may further enhance HIF-1α signaling. After nuclear translocation and dimerization with HIF-1β, HIF-1α regulates downstream programs involved in angiogenesis, metabolic reprogramming, EMT/invasion/metastasis, stemness maintenance, dedifferentiation, and RAI resistance, and stress adaptation/microenvironment remodeling. These changes collectively contribute to thyroid cancer progression, therapeutic resistance, and poor prognosis. Solid borders denote thyroid cancer-specific evidence, whereas dashed borders denote broadly shared hypoxia-associated mechanisms.

Collectively, these findings indicate that hypoxia influences thyroid cancer behavior through multiple interconnected mechanisms, as summarized in Figure 1 and Table 1.

## 4. HIF Inhibition as a Potential Therapeutic Strategy in Thyroid Cancer

Available preclinical evidence suggests that HIF signaling is a relevant therapeutic target in thyroid cancer. In thyroid cancer models, HIF-1α inhibition has been reported to reduce VEGF expression and suppress tumor cell proliferation and migration [31]. The novel HIF-1α inhibitor IDF-11774 also suppressed thyroid cancer cell proliferation and glycolysis by inhibiting HIF-1α-dependent transcription [31]. Together, these findings support the biological relevance of HIF inhibition as a potential therapeutic strategy in thyroid cancer, although current evidence remains largely preclinical.

PX-478, a well-characterized HIF inhibitor, has shown antitumor activity in preclinical models and has undergone phase I clinical evaluation in patients with advanced solid tumors (NCT00522652). However, direct evidence for PX-478 in thyroid cancer remains limited. Although its ability to suppress HIF-1α synthesis suggests potential relevance for aggressive thyroid cancer subtypes, this possibility has not yet been validated in thyroid cancer-specific clinical studies. In addition to direct HIF inhibition, some targeted therapies currently used in thyroid cancer may also modulate hypoxia-related pathways. For example, lenvatinib, a multikinase inhibitor approved for radioiodine-refractory differentiated thyroid cancer, inhibits VEGFR and fibroblast growth factor receptor (FGFR) signaling and may influence hypoxia-related signaling within the tumor microenvironment [114]. Overall, interventions targeting HIF-1α and its downstream angiogenic pathways may represent a potentially valuable therapeutic direction in thyroid cancer, particularly in the context of dedifferentiation and treatment resistance; however, further thyroid cancer-specific translational and clinical validation is needed. In addition to direct inhibition of HIF-1α, the broader field of translational research includes a number of representative hypoxia-related strategies that are not yet established in the field of thyroid cancer. HIF-2α-targeted agents such as belzutifan illustrate the clinical feasibility of hypoxia-pathway targeting in non-thyroid solid tumors. In contrast, redifferentiation approaches such as selumetinib plus radioiodine remain clinically relevant to thyroid cancer because of their close relationship to dedifferentiation and radioiodine-refractory disease [115,116].

To improve the translational relevance of this section, selected hypoxia-targeted or hypoxia-relevant therapeutic strategies, along with their current clinical or translational status in thyroid cancer, are summarized in Table 2.

## 5. Implications for Diagnosis, Prognosis, and Therapy

Traditional prognostic assessment in thyroid cancer is based largely on clinicopathological factors such as tumor stage, tumor size, lymph node involvement, and driver mutations. However, these models do not always fully capture tumor heterogeneity, biological aggressiveness, or treatment resistance. Because HIF-1α expression and other hypoxia-associated features have been linked to dedifferentiation, invasiveness, and metastatic potential, hypoxia-related markers may provide additional value for refining current diagnostic and prognostic frameworks.

In PTC, elevated HIF-1α expression has been associated with adverse clinicopathological features, including extrathyroidal extension, LNM, and advanced TNM stage [28,90]. A recent meta-analysis further supported the association between HIF-1α overexpression and more aggressive disease characteristics in PTC [28]. Similarly, Zhang et al. [30] reported that co-expression of HIF-1α and caspase-3 in aggressive PTC variants was associated with shorter disease-free survival and higher relapse rates. In addition to tissue-based markers, circulating hypoxia-associated factors may also be clinically relevant. Li et al. [117] showed that serum HIF-1α and HIF-2α levels were significantly elevated in patients with FTC compared with those with benign nodules and were independently associated with vascular invasion and increased recurrence risk. Their receiver operating characteristic (ROC) analysis suggested that serum HIF-1α may have potential utility for identifying higher-risk FTC. Taken together, these findings suggest that hypoxia-related biomarkers may complement histopathological assessment in identifying patients at greater risk of aggressive or recurrent disease, although further validation is needed.

Beyond individual markers, transcriptome-based hypoxia scores may provide a broader measure of intratumoral hypoxia and its clinical relevance. Ma et al. [118] demonstrated that in PDTC and ATC, higher hypoxia scores were associated with greater genetic instability, lower differentiation status, and shorter survival. Integration of hypoxia-associated gene expression profiles with clinicopathological and molecular features may therefore improve risk stratification, particularly in patients with BRAFV600E-positive or radioiodine-refractory disease.

Hypoxia-related pathways may also have therapeutic implications. As discussed above [31], preclinical studies suggest that pharmacological inhibition of HIF-1α may have therapeutic potential in thyroid cancer. These findings suggest that HIF-1α may represent both a biomarker candidate and a potential therapeutic target in high-risk thyroid cancer. However, the therapeutic relevance of HIF-targeted strategies in thyroid cancer remains to be established in disease-specific translational and clinical studies.

## 6. Conclusions

Hypoxia is an important feature of the TME in thyroid cancer and is increasingly recognized as a contributor to tumor progression, dedifferentiation, metabolic adaptation, stemness maintenance, angiogenesis, and treatment resistance. Available evidence suggests that HIF-associated signaling plays a central role in linking hypoxic stress to aggressive tumor behavior, particularly in PDTC and ATC. At the same time, the strength of evidence varies across biological processes and thyroid cancer subtypes.

Despite certain progress, several important questions remain unresolved. Much of the current evidence is derived from cell-based experiments, retrospective tissue studies, or preclinical models, and further thyroid cancer-specific clinical validation is needed. Future studies should clarify the clinical relevance of hypoxia-related pathways across thyroid cancer subtypes and determine whether hypoxia-directed strategies can provide meaningful benefit in advanced or radioiodine-refractory disease. A better understanding of hypoxia in thyroid cancer may improve risk stratification, help identify patients at increased risk of aggressive or treatment-resistant disease, and support the development of more personalized therapeutic approaches. In addition, emerging hypoxia-imaging approaches, particularly PET-based tracers such as 18F-FMISO, 18F-FAZA, and 18F-HX4, may further improve the precision of hypoxia-directed management in thyroid cancer. These modalities may enable noninvasive assessment of intratumoral hypoxia, support patient stratification, and help identify tumors with more aggressive biology or reduced sensitivity to radioiodine therapy. In this context, molecular imaging of hypoxia may also facilitate the future clinical development of hypoxia-targeted or redifferentiation-oriented therapeutic strategies [84,119,120].

## Figures and Tables

**Table 1:** Principal molecular mechanisms by which hypoxia regulates thyroid cancer behavior.

Functional Category	Molecule/Signaling Pathway	Mechanism of Action	Impact
Angiogenesis	VEGF	Promotes tumor angiogenesis	Enhances tumor growth, invasion, and metastasis [34,40]
EMT/metastasis	PI3K/Akt/GSK3β SIRT6/HIF-1α	EMT	Facilitates tumor metastasis [36,37]
Stemness maintenance	HIF signaling/CSC maintenance	Maintains stem-like phenotypes and aggressive behavior	Promotes tumor progression and metastatic potential [72]
Dedifferentiation	miRNA-210	Promotes tumor dedifferentiation	Enhances tumor growth, invasion, and metastasis [80]
Therapeutic resistance/RAI resistance	HIF-1α/YAP HIF-1α/β-catenin PKM2/NF-κB	Upregulates GLUT expression, downregulates NIS	RAI resistance [62,87,88]
		downregulates NIS	
		Promotes resistance-related inflammatory and metabolic signaling	
Additional hypoxia-responsive pathways	HIF-1α/FGF11 HIF-1α/TERT	Upregulates FGF11 Induces autophagy	Enhances tumor growth, invasion, and metastasis [112,113]
ECM remodeling/biomechanical adaptation	ECM remodeling/matrix stiffness	Promotes stromal remodeling, collagen cross-linking, and altered tumor–stroma interactions	Facilitates tumor invasion, therapeutic resistance, and altered radiation response [19,96,97]
Stress adaptation/therapeutic resistance	UPR/heat shock response	Maintains proteostasis and reduces apoptosis under hypoxic stress	Enhances tumor cell survival and may contribute to radioresistance/drug resistance [106,107]

**Abbreviations:** HIF, hypoxia-inducible factor; VEGF, vascular endothelial growth factor; PI3K, phosphoinositide 3-kinase; Akt, protein kinase B; GSK3β, glycogen synthase kinase 3 beta; EMT, epithelial–mesenchymal transition; SIRT6, sirtuin 6; YAP, Yes-associated protein; GLUT, glucose transporter; NIS, sodium/iodide symporter; CSC, cancer stem cell; miRNA-210, microRNA-210; NF-κB, nuclear factor kappa B; FGF11, fibroblast growth factor 11; TERT, telomerase reverse transcriptase; ECM, extracellular matrix; UPR, unfolded protein response; RAI, radioiodine.

**Table 2:** Selected hypoxia-targeted or hypoxia-relevant therapeutic strategies and their current clinical or translational status in thyroid cancer.

Strategy/Agent	Target or Rationale	Study Level/Evidence Type	Relevance to Thyroid Cancer	Current Clinical/Translational Status
IDF-11774	HIF-1α-targeting small molecule that suppresses HIF-1α-dependent transcription and glycolytic adaptation	In vitro thyroid cancer evidence (Preclinical)	Directly relevant to thyroid cancer cell models	No thyroid cancer-specific clinical trial identified
PX-478	Direct HIF-1α inhibitor	Phase I clinical evidence in advanced solid tumors	Mechanistically relevant to aggressive thyroid cancer, but no thyroid-specific cohort has been reported	Evaluated in a completed phase I solid-tumor study; thyroid cancer-specific clinical evidence remains lacking
Belzutifan	Direct HIF-2α inhibitor	Clinical evidence in non-thyroid solid tumors	Mechanistically relevant to hypoxia biology, but not yet established in thyroid cancer	Current clinical development is concentrated mainly outside thyroid cancer
Hypoxia PET imaging (e.g., 18F-FAZA PET/CT)	Noninvasive assessment of tumor hypoxia for risk stratification and treatment guidance	Exploratory translational evidence in thyroid cancer	Directly studied in metastatic thyroid cancer	Exploratory evidence suggests tumor hypoxia may be associated with short-term progression after radioiodine therapy
Redifferentiation/RAI-restoration strategies (e.g., selumetinib + I-131)	Clinically relevant to hypoxia-associated dedifferentiation and radioiodine resistance, although not a direct HIF inhibitor	Phase II thyroid cancer evidence	High relevance to refractory thyroid cancer	Thyroid-cancer-specific clinical evaluation is available

**Abbreviations:** HIF, hypoxia-inducible factor; RAI, radioiodine. **Note:** Because thyroid cancer-specific clinical trials of direct HIF inhibitors remain very limited, representative solid-tumor clinical studies and thyroid cancer-relevant translational strategies are summarized together.

## Data Availability

No new data were created or analyzed in this study.
